# Compressed Air

**Published:** 1906-03-15

**Authors:** Geo. Zederbaum


### COMPRESSED AIR.

BY GEO. ZEDERBAUM, D.D.S.

Read before the Detroit Dental Society, November 9, 1905.

At the time I received Dr. Giffen's request to read a paper on compressed air and pressure anesthesia, I felt a good deal, no doubt, like many of you have felt, when, during the quiz hour, the professor unexpectedly called your name, and, much to your further surprise, propounded a question you knew how to answer; so it was in my case. Compressed air is a pet hobby of mine; and some of you, I know, heard me singing its praises, at Lansing in June, 1904.

Compressed air for dental purposes is no more a novelty. A well equipped office of this day, in addition to its fountain cuspidor, sterilizer, electric engine, electric motor-lathe and porcelain oven, has also its compressed air supply. I do not regard an office as well equipped in every sense of the word, if this, to many minds a luxury, is absent.

I need not go into detailed description of any of the compressed air appliances on the market; as the selection of one depends on individual opinion, on location, on extent of practice and on whether mostly prosthetic or the operative branch.

On individual opinion—inasmuch as you are apt to consider articles of a certain manufacture better than others. On location—because it is not in every town you can get the street current all day, and storage batteries are not of much value. On extent of practice—because I do not approve of a beginner going into equipment beyond his means, nor beyond what his limited practice would permit at the outset; and on extent of operative practice as compared with crown and bridge work, because either of these would, in my opinion, call for a diffeıent outfit.

In my practice I use the compressed air mostly at the operating chair foi the utility of compressed air at the work bench is comparatively small. Of course it does away with the bellows—and those of you who are not acquainted with the uses of compiessed air don't know with what ease you can do your even extensive soldering. I find it also very useful for gas or gasoline furnaces and also for cooling warm plastic impression material while in the mouth which alone is a desirable feature.

The applications of compressed air for the operating room are by far more numerous. I will mention them in the order of their respective usefulness. At the outset, I wish it understood that I am a stern advocate of rubber dam; but, gentlemen, all of you have met cases where it could not be placed at all; cases where it could not be placed advantageously, due either to a mal-shaped tooth or a mal-posed one. Often asthmatic or catahrral conditions, prevalent here in Michigan, preclude the use of dam; and it is just for such cases that the compressed air becomes invaluable. Here you use a current of air as warm and as strong as the patient can bear comfortably; and, in a few minutes, you have not only made the tooth and surrounding tissues absolutely dry, but you have also practically anesthetised them by dehydration. You have prepared your cavity in the same manner, and under the best conditions for placing your filling material; and, what is also important, you afforded ease and comfort to your patient. Many would suggest the saliva ejector for such cases, or cotton rolls, etc., but gentlemen, in my hands the saliva ejector has proven for most mouths but a poor excuse; and as for cotton rolls, well, I'd rather not have them, and I think that the patients would also bear me out on that point.

Another practical use of compressed air is the constant blowing out of chips while excavating and drilling cavities. I often invite my patient's co-operation and he will not only keep the field of opeiation dry, but will also have it clear of all chips and debris and will minimize his pain by having his mind on his share of the work.

Before going further, I wish to say a few words in favor of heated air. I have yet to meet a patient who did not find immediate relief while the preparation of some sensitive cavity was done under dry conditions brought about by heated air. Warm air for obtunding hypersensitiveness of dentine was used for many years; but not until the last few years has it become so effective and so generally used, and simply because we never had a continuous strong stream of air until we got to using compressed air outfits. We also have better ways of heating the air, and we run less danger of burning the soft tissues, because we need not have the nozzle in the cavity, a few inches away will do better, and the air coming as it does in one continuous stream does not get cooled enough before reaching the cavity to retard the desired effect. One should never attempt to employ the compressed air at a higher temperature than can be comfortably borne on the back of ones hand. Always start in slowly and do not direct the current immediately at the point to be operated upon, but a little away at first, closing in gradually until you are sure you have accustomed the tissues to it sinfluence and have dispelled your patient's possible fright and thereby perhaps avoided some disastrous result. Here we must be even more careful than when soldering a porcelain bridge—here, we deal with a live subject capable of responding even with fistic demonstrations.

Other invaluable uses of compressed air in dentistry are: Its application in prophylactic treatment, in cleansing the teeth, and in treating pyorrheal pockets. This is a prophylactic age, and here, there and every where we hear of prophylactic measures calculated towards bettering the general conditions in the mouth. I regard a thorough cleansing of teeth and massaging of gums as one of the most important operations we are called upon to perform, and I charge accordingly. Along with the scalers I employ the compressed air for two purposes:—

First, with a fair degree of pressure directed into the embrasures and about the necks of teeth I am better enabled to see the location of calcareous and other deposits; and when you can see, you don't have to feel and hunt for them. (Dr. Hoff might take issue with me on that point, but I am sure it requires considerable practice to rely entirely on the sense of touch, and there are very few of us who attain that great degree of fineness.) Compressed air actually blows aside the naturally inflamed gum and thereby enables one to remove all deposits with greater accuracy.

Secondly, after scaling, by means of the compressed air nozzle attached to a common atomizer, you can spray the gums rootward as deeply as needed, even to the bottom of pyorrheal pockets. Either a dry or a liquid medicament works perfectly. In treatments of antral empyema, or wounds from root excision or extraction, you can force medication to the deepest portions and do more good and in a shorter period than by the old methods. One can easily control a secondary hemorrhage by spraying emptied root sockets with Adrenalin solution with a fair degree of pressure. Powdered styptics can also be used in similar manner.

In setting crowns and bridges, we ought to have absolute dryness, and we can have it with the compressed air apparatus. Furthermore, I will say, that after the pressure has been used a few minutes, it will not only dry the abutments and piers, but actually numb the gum tissue and lessen the pain incidental to placing shell crowns. I have tried the air on my own mouth and found this to be true to a great extent. Did you ever attempt to set a lower bridge, when the saliva completely covered the abutments? Did your bridgework stay? With the compressed air properly used, you need not anticipate another loose piece of work to re-cement within a day or so.

How about deodorizing malodorous mouths before any operation? Occasionally (but only occasionally, thank heaven), you get a patient in the chair, and the minute you get a smell of his breath you wish you were in the proverbial "high-hole's" nest. This type usually excuse themselves by mentioning they had not cleaned their teeth that morning! I wish to ask whether it is healthy for us to inhale the foul air that emanates from such mouths. Why not use some deodorant? I use a strong aqueous solution of oil of cassia, spraying the mouth several times, until the breath be at least somewhat improved.

While touching upon deodorization, I wish to repeat what I have said many times before, that is, try to keep your operating rooms free from the smell of drugs. Patients come into dental offices with a degree of fear which is increased the the second they smell drugs in the operating room. It is my habit to spray this room with spirits of lavender. Compressed air does it in a minute, and the air is freshened and decidedly more agreeable.

After nitrous oxide administration, your patient can be brought to quickly by a force of air striking squarely in his face—a fresh breath is caught and the patient returns to consciousness.

Local anesthetics are very desirable—but many object to the hypodermic needle. If this objection is not overruled, I use ether or ethyl chloride, and, by means of compressed air, freeze the gums quicker and more effectively than I used to do it without the compressed air outfit.

Gentlemen, I fear that my paper will be too long if I enumerate many more ways of utilizing the compressed air in dental offices. There are many more, and every few months I learn of others. I cannot say that it will do everything in every case, no, not any more so than any other apparatus we employ—but it does so much good that I firmly believe it only a matter of time until every modern office will have a system of compressed air for both the operating room and the laboratory. And who knows, perhaps it will come to pass that some day we will use the compressed air in connection with pressure anesthesia.

### DISCUSSION.

DR. HAMPTON: Compressed air is a very interesting subject. It has come to stay. It is not a novelty that will be here but a little while. In regard to pain caused or relieved by the compressed air, I believe the effect is produced largely by variation of force and temperature. It is much the same as with an electric current: taking hold of a battery current that flows steadily one can feel no current, but as soon as we get a break in the current, as we get in the vibrator, we feel it immediately, it is the change of current that causes the shock. With the air current if we get a varying force a great deal of pain is caused, while if we have a steady pressure with the regulator on the switchboard to tell just how much pressure we have, by maintaining it evenly on or near the tooth, we can get the same results that we can with the chip blower.

In regard to reaching the pockets of the tooth, I think it is the best way to remove the loose tartar; after scaling in the pocket the tartar must be removed and we cannot get it all out with the scaler, because it will not grip to it. The blowing of the air into the pockets will in a great many cases help a great deal to relieve the irritation that is caused by these little pieces of tartar remaining loose inside the pockets.

Dr. Graham told me the other day that he uses the compressed air when polishing a gold filling. I can see where it would be splendid in reducing the temperature and making the work more rapid, and also be very grateful to the patient. In this either the assistant or the patient himself could aid in holding the air nozzle. In Dr. Graham's office I saw how he utilized the compressed air in heating a bridge for soldering. He uses the idea of the Jenkins furnace where the gas comes from below, and uses the protection about the same general shape as the Jenkins protector, there is a slot in the side through which he can work and move the case around. The blow pipe is used through the top while a steady current is coming from below which he can regulate. By using the compressed air for the blow pipe, we can readily see how, with the compressed air, we can get a steadier temperature and have less trouble in checking the porcelain.

Dr. Fechheimer told me of an apparatus that he uses, or saw, in which a steady stream of water of varying temperature can be used in connection with compressed air. It is a device by which, on placing the thumb over a little opening in the tube, you can vary the amount of water which comes through, and by another pressure vary the temperature, so that you can get a steady flow, much or little, and of varying temperature as you wish it. With a stream of water of proper temperature flowing into the cavity while you are drilling the pain can be greatly diminished.

I saw a few years ago an apparatus electrically supplied for heating water in much the same way. As the water came through a tube there is a coil of platinum around it which heats the casings, and they warm the water as it comes through. Under the regular flow from the faucet it would shoot a fine stream into the cavity. I saw a dentist with one of these drill out a cervical cavity and the patient sat still, smiling most of the time.

I do not like to think of using compressed air in the treatment of antrum cases as Dr. Zederbaum suggests. In these cases the least pressure that can be used in clearing out and forcing medication in, the better.

Dr. Loeffler: Don't you know that the thing that appeals to the public more than anything else is how easy you can make the work for them? It is sometimes humiliating when a patient comes to us and says "I heard that you were very gentle and had all sorts of things to make operations easy." Why don't they inquire about our skill? They take it for granted, nowdays, that any dentist has plenty of skill. I think the compressed air advocated is a suggestion that does this very thing—has a tendency to make operations easy for the patient and for ourselves. We all want appreciative patients and we want to get the reputation of trying to do away with pain as much as possible, sufficiently so that people will come to us again.

You can even have a hand pump that you can work yourself, and you will be surprised how much air you can use during the day under pressure. Don't do without it simply because you have not all the automatic contrivances suggested.

In regard to the test of temperature, I have found the test on the side of the nose to be a very sensitive one, and it presents it nearer to the field where you will use it. Lately I had a case of a very severe necrotic alveolar abscess in the lower jaw, where, by using a steady current of air, I was able to see down to the bottom of the pocket and to observe what was going on. What you can see with your eyes appeals to you much better than with your sense of touch.

When you begin a surgical operation you can use some cheap alkaline solution if you wish, and it will answer very well to cut the mucous and make the mouth fit foı the operation. When you get through, nothing is so pleasant, even after a serious and long operation, as to spray with some mouth wash, as boral or glycothymoline, at the proper temperature, this always has a pleasant effect upon the patient.

In regard to pressure would say that I have a tank pressure of thirty to forty pounds, but I seldom use more than fifteen pounds. For drying out cavities or where I want only the warm air, with the gage on the switchboard I run it down to a pound or so.

DR. ZEDERBAUM: The water motor is something we can use almost every where, while the electric device we cannot have in every place. However, where you have a water motor you are obliged to put in some other apparatus for the hot air part of it. Without the hot air you cannot do all the good that compressed air can do. Therefore you must have a Perry hot air syringe or a battery to run a special electric syringe such as is made by Gerhart also.

Regarding the pressure necessary, I find that more than fifteen or twenty pounds is not necessary, although where you have a regulating device it can be turned on as the pressure decreases later in the day. Too strong a force a patient will shrink from.

About using compressed air while polishing gold fillings that is a very good idea. I also recommend it while drilling cavities any place where the burr is apt to become overheated. Also in hollow porcelain crowns, the tooth becomes overheated, especially where you have a live nerve to deal with, and the patient can assist you very materially by keeping the pressure on and cooling the point of the operation, you can work more rapidly and do better work

Regarding the stream of hot water, while it is a known fact heat is good, hot water I would consider objectionable in some cases. Where you use the rubber dam you use it to keep out the moisture. Hot air without the water will do just as much good as hot water.

In scaling, especially where we have a considerably inflamed condition of the gums, it is almost impossible to scale thoroughly and not cause hemorrhage of the gums. This is where compressed air is decidedly a great advantage, the blood is kept away and you do not have to stop every second to let them wash it out. In hemorrhages after extracting, especially in secondary hemorrhage, the placing of plugs is very painful and not very satisfactory at best, inasmuch as you have, after a short time, to pull that plug out. There is where you can use compressed air with an atomizer point. You can place the dry medicament where you want it and can stop the hemorrhage much better.

In fainting spells it is often useful. Aromatic spirits of ammonia is a good thing, but many times you cannot give it and they do not breathe quick enough to get any benefit of the smell, so spray some of it into the nose through the atomizer and they will feel it in a minute.

There are many uses for compressed air. One use is to dry your hypodermic syringe needle and keep it free from obstructions. Compressed air will do this; insert your needle point into the nozzle. You can put some vulcanized rubber over the nozzle and it will blow it right through.

I read in the *Scientific American* where they used compressed air for mining, drilling, uprooting gnarled oaks. It is possible that we may use compressed air to extract teeth yet.

I wish to say a word regarding the treatment of empyema. Dr. Hampton asked me to mention a few facts if I knew of any, and a special point for a minute will be on compressed air in connection with that. We know that, as nearly as I can remember, ten or fifteen years back a majority of the best rhinologists and surgeons used only atomizers with rubber bulbs, and the squeezing of the bulb did not prove effective as the current was always interrupted. I remember distinctly ten or twelve years ago I had a sore throat and the surgeon then had a tank something on the principle described here to-night. He pumped the tank full by hand power and used air from it in connection with his atomizer. My throat got well and I do not believe my sensitive tissues suffered much either. Nowadays we know most of the big buildings for doctors' offices, in the City of Chicago, are supplied with compressed air, and they use that almost exclusively in spray work of all kinds. You do not have to use it forcibly; use it just as light as you need it and you still have a better effect, for the medicament will have a continuous flow and you can get deeper penetration. The trouble with empyema of the antrum is almost invariably that there are other complications about the nose, and the patients call a rhinologist. In this disease the pus cavity must be emptied, usually through the meatus. If it gets through at all it makes as much of an opening as is necessary and no great trouble ensues. Not long ago I read an account of how the Prince of Wales being affected with this disease, and the rhinologist made an opening but did not succeed in draining the cavity as well as it should be; at least he was obliged to order the Prince to stand on his head in the corner for five minutes to drain it. The general surgeons invariably make their opening for empyema through the canine fossa. They believe this is the proper place to empty it, but these openings many times do not heal as readily as the operation that dentists advocate, which is to enter between the buccal roots of the first and second molars. If your first and second molars are bad, extract them, and then there is no trouble in making the opening and draining the cavity as thoroughly as can be. If, however, the teeth are sound, make the entrance between the buccal roots of the first and second molars.

N. K. GARHART: Compressed air is used for a great many operations in dentistry, such as treating sensitive dentine by placing a preparation of cocain on cotton, placing it in the cavity and driving it into the tubuli by means of a pressure of hot air, first applying air of a luke warm temperature and gradually increasing the heat so that the tooth will become accustomed to the thermal changes which take place. Again it is very useful in drying out root canals. It is an established fact that a stream of compressed air will completely dry out the root canal without having to pass the point well up into the root of the tooth. Of course it's chief use is the drying out of cavities, and I must say that no other method affords such good results as that of a constant stream of air, well heated, and under a pressure of say from ten to twelve pounds. If the pressure is too high, it is quite annoying to the patient, therefore it is not advisable to use a higher pressure than the amount stated above.

In putting in a compressed air outfit, I would advise that the dentist be fully informed as to the necessity of obtaining the right kind of appliance, so there will be no future trouble in the way of experiencing leaks in the valves of the line connections between the pump and the tank, also the tank and switchboard. There is only one kind of valve, which, from the standpoint of experience, will continually hold up under a pressure of compressed air without leaking, and this is what is termed a "needle point" valve. However, it has the one disadvantage of having to be repacked occasionally. This valve carries what is known as a "stuffing box" in order to prevent, leaks along the stem. All the valves used between the switchboard and tank also tank and pump should be of this type.

The tank should be made of heavy material capable of withstanding four times the highest pressure which it is supposed to carry. The best one for this purpose is an ordinary hot water heater such as is used in dwellings. However, these stock tanks come with four or five 1 inch holes in them, and these, with the exception of two, must be plugged with ordinary plugs for the purpose. In order to secure air-tight joints, it is necessary to wipe the threads of both, hole in the tank and the plug, with white lead, after which the plug should be screwed tightly into place. The two top holes on the upper surface of the tank are to be fitted with metal fittings and attached to each end should be a *needle point* valve. These fittings when screwed together must be calked by the use of white lead as stated before.

The pump should be directly connected with the tank using lead tubing for this purpose. The joints should be soldered and the tubing which leads from the switchboard to the tank should also be soldered as well as from the pump to the tank. Having equipped your office with a compressed air apparatus of this kind, there will be no trouble in the future to keep the tank pressure up, and at the same time, there will be no leaks in the line after the apparatus has been in use for a long time.

In regard to the tank, I would further state that there are two kinds on the market. The one having the top and bottom riveted in, is the most satisfactory for your purpose. As to the choice of pumps, will say that it does not matter whether you use an electric motor driven pump or the kind known as the water pump. Both give very satisfactory results, although there is some advantage in favor of the water pump, because of its automatic features. That is to say, the water pump operates upon a principle that, when the pressure in the tank is equal to that of your water supply it naturally causes the pump to stop and as soon as the pressure in the tank becomes lower than that of the water supply, it starts the pumping. However, this pump requires packing at least once a year although the cost of this work does not amount to over $2.00 to $3.00, I certainly would not advise the use of the water pump in cities where the supply of water is muddy or filled with sediment, owing to the fact that the solid matter has a tendency to wear out the packing in the pump, necessitating frequent repairs. The motor driven pump does not get out of order, although it does not possess the automatic feature as will be found in the water pump. In other words, when starting the pump, you will have to watch it until the pressure in the tank is of 50 pounds, and then tuin it off again. You will also have to start the pump when the pressure is run down in the tank. There are a few automatic switches on the market that control the operation of the pump. However, the cost of the pump and this automatic switch is almost once again the price of a large water pump.

It is advisable to have a pressure of say 50 pounds in the tank which affords a copious supply for operating and prosthetic work. You will understand that compressed air in the laboratory is very useful, especially for crown and bridge work. However, a pressure of fifty pounds is too great to be used in either the laboratory or operating room, so you will have to provide a pressure of say from ten to twelve pounds. There can be had from various concerns small air regulators which do this work very nicely. By means of turning a knob on such an appliance, you can set the pressure anywhere from one to fifty pounds. The various switchboards which are sold on the market, those which are complete in eveiy detail, cariy a regulator for this purpose, also two gauges one of which indicates the pressure of the tank supply, while the other indicates that pressure which is reduced by the regulator.

In regard to appliances for use of compressed air in the operating room, will say that the hot air syringe and the atomizers constitutes a complete equipment. With the hot air syringe you may use either a hot or cold blast of air. The syringe which we manufacture will give either hot or cold air by pressing on the small lever on the handle of the syringe. When the lever is full down, hot aii is formed; when partly down, cold air is pioduced. The temperature of the hot air is modified by intermittent contact of the lever. Such a syringe is absolutely automatic in action, for when the lever is released, the compressed air and electrical current supply are cut off automatically.

In regard to the atomizers, they are especially desirable for tieating cases of pyorrhea. By means of such an instrument, the medicament can be applied to the pockets in the form of a spray with the absolute certainty of medicating the parts thoroughly.

Again we have on the market small gold needles of an angle shape similar in size to those used for hypodermic syringes. The purpose of these needles is to insert into the root canal for the purpose of applying antiseptic treatment.

The various manufacturers of electric appliances also put on the market a heater for keeping the spray bottles warm. The use of compressed air has a tendency to chill the solution which is not very comfortable to the patient.

